# Prevalence of leptospirosis among patients attending renal and general outpatient clinics in Mulago Hospital, Kampala, Uganda

**DOI:** 10.1038/s41598-022-12544-3

**Published:** 2022-05-19

**Authors:** Rogers Wambi, William Worodria, James Muleme, Siya Aggrey, Lawrence Mugisha

**Affiliations:** 1 Mulago Hospital, Kampala, Uganda; 2 College of Veterinary Medicine, Animal Resources & Biosecurity (COVAB), Makerere University, Kampala, Uganda; 3 EcoHealth Research Group, Conservation & Ecosystem Health Alliance (CEHA), Kampala, Uganda; 4 Department of Disease Control and Environmental Health, School of Public Health, College of Health Sciences, Makerere University, Kampala, Uganda; 5 Department of Zoology, College of Natural Sciences (CONAS), Makerere University, Kampala, Uganda

**Keywords:** Microbiology, Environmental sciences, Diseases, Health care, Health occupations, Pathogenesis, Risk factors

## Abstract

In this study, we sought to establish the prevalence of leptospirosis among renal patients and general outpatients attending Mulago National Referral Hospital, Uganda. A total of 254 patients were recruited, their blood samples collected and interviewer-administered semi-structured questionnaires provided between July and October 2018. These questionnaires captured data on sociodemographic characteristics and symptoms of leptospirosis disease. An individual with an average body temperature of 37.3 ± 1.1 °C was considered to be having fever. The blood samples were analyzed using the standard Microscopic Agglutination Test (MAT) with a panel of 14 *Leptospira*-serovars belonging to 11 serogroups. Prevalence was reported with confidence intervals while questionnaire data was analyzed using logistic regression analysis. We present an overall prevalence of leptospirosis at 4.70% (95% CI = 2.60–8.30) after analysis of samples from recruited patients. This seropositivity (12/254) was classified into 7 serovars, among which, *Canicola* and *Djasiman* presented with titers between ≥ 200 and ≥ 400 in samples of both renal patients and outpatients, indicative of the active disease. *Djasiman* was the highest contributor to the reported prevalence. Overall, most examined participants presented with common symptoms of abdominal pain (AOR = 24.4, 95% CI (2.42–267.89), *p* = 0.02) and dehydration (AOR = 0.1, 95% CI (0.01–0.69), *p* = 0.05). Our study suggests that these symptoms and previous history of abdominal pain may be caused by *Leptospira* infections among the studied participants. We therefore recommend inclusion of leptospirosis in the differential diagnosis for renal and febrile illnesses. Indeed, abdominal pain and dehydration should be further studied with a bigger sample size and for other related febrile illnesses.

## Introduction

Leptospirosis is a zoonotic infection caused by bacteria of genus *Leptospira*^1^. It is considered one of the world’s public health challenges due to its greatest spread at global level^2^. Studies have indicated that domestic animals (dogs) as well as wild animals (rodents) are great reservoirs for this disease causing bacteria^3,4^. Infected animals are able to shed the *Leptospira*-spirochetes (being distinguished by the helical shape of the bacteria) through their urine into the environment where other hosts easily pick them^1^. These *Leptospira*-spirochetes in addition have the ability to withstand harsh environmental conditions such as pH and temperature^5^. This ultimately accounts for their wide geographical distribution and increased chances for new host infections worldwide. Indeed, estimates at global scale indicate 1.03 million cases and 58,900 deaths occur annually as revealed by Leptospirosis Epidemiology Reference Group (LERG)^6^. Additionally, various studies across Sub-Saharan Africa (SSA) reveal a (2.3–19.8)% prevalence of acute leptospirosis among febrile patients who attend healthcare facilities^7–9^.

In Uganda, studies that have been conducted among dogs and humans reveal a leptospirosis prevalence of 27% and 35% respectively, using Microscopic Agglutination (MAT) test^10,11^. Like it is across Africa, the human patients diagnosed with leptospirosis normally present with fever during their visits to the health facilities^12^. Despite this documented burden of leptospirosis, its febrile nature makes the disease to be misdiagnosed, under or un-diagnosed especially in resource constrained healthcare settings^13^. Indeed, late detection of this bacteria may lead to complications associated with progression of the infection into complicated stages of multi-organ involvement such as kidney and lung dysfunction^14–16^. It is important to note that health care facilities and hospitals tend to diagnose causes of febrile illness using blood culture techniques with media that does not support growth of *Leptospira* thus ending up missing leptospirosis diagnosis. For instance, majority of the samples from febrile patients submitted for bacterial blood culture usually turn out to be negative for any infectious organism according to the laboratory records and other published literature about Mulago Hospital^17^.

Adequate data on the prevalence, signs and symptoms associated with leptospirosis would facilitate the design of diagnostic algorithms for use in resource limited settings. This would be important in SSA countries like Uganda where healthcare systems are fragile to allow for early detection of such diseases. Therefore, our study sought to establish the prevalence of leptospirosis as well as ascertaining the signs and symptoms related to the infection among patients attending renal and general outpatient units in Mulago National Referral Hospital, Uganda.

## Materials and methods

### Study design

This cross-sectional study was conducted between July and October 2018 among renal and general outpatients within Mulago National Referral Hospital (MNRH). The study utilized a quantitative approach which involved blood sample collection from patients and administration of a semi-structured questionnaire to collect data on the patient factors, signs and symptoms.

### Study site

Uganda’s only national referral hospital, MNRH has approximately 2072 health care professionals as of the (*Human Resource for Health Audit Report 2017*). The hospital receives referred patients from all districts of the country with official capacity of 1790 beds, although it often houses over 3000 patients.

The hospital has an established renal clinic running every Tuesday of the week. Patients with renal problems are required to present a proof of diagnosis from either the general outpatient of MNRH, or any other private or public healthcare facility. Patients in the general outpatient unit on the other hand normally walk-in with their referral letters from lower-level government or private healthcare facilities even to some extent patients arrive without any medical records (self-referral).

### Study population

The study population comprised of both female and male participants with the age range of (5–87) years attending renal and general outpatient units in Mulago hospital Kampala (Uganda) during the study period. The patients in this study presented with febrile illness or renal dysfunction as sampled from the general outpatient and renal units respectively.

During the study period, the renal unit received a total of 180 patients and of these, only 119 agreed to participate in the study. Following the inclusion criteria, only 135 patients from the general outpatient unit qualified and consented to voluntarily participate in the study. Overall, sample size of 254 (119 and 135) patients were included in the study.

### Data and sample collection procedures

#### Data collection

A structured and pre-tested questionnaire was administered to all the 254 participants recruited to the study. This data collection tool was used to obtain information about the patient, signs and symptoms among others (Details of the data collection tool refer to Supplementary I).

Our study also collected other patient’s information such as body temperature, physical examination and history of previous treatment as assessed by the clinician and filled special laboratory request form (Details of the laboratory request form refer to Supplementary II). Additionally, the urine color (dark-dehydration) and (light for hydration) suggested the hydration status of the patient. An individual with an average body temperature of 37.3 ± 1.1 °C was considered to be having fever.

#### Blood sample collection

Blood samples were collected from participants and allowed to stand for between 30 and 60 min to enable clotting. This was then centrifuged for 5 min at a speed of 3000 rpm centrifugal force to separate the serum. Serum was then aliquoted in to cryovials, packed in the cool box and transported to Central Diagnostic Laboratory (CDL) at the College of Veterinary Medicine Animal Resources and Biosecurity, Makerere University for analysis.

### Laboratory procedures

During testing at CDL, the standard microscopic agglutination test (MAT) with a panel of 14 *Leptospira* serovars belonging to 11 serogroups was used. All serovars used were maintained in Ellinghausen–McCullough–Johnson–Harris (EMJH) liquid media and sub-cultured weekly to maintain the live serovars panel. Cultures which were 5 days old and showed some growth of *Leptospira* under dark-field microscopy were selected for use. These serogroups with their respective serovars included were: *Autumnalis* (*L. interrogans* serovar *Autumnalis*), *Ballum* (*L. borgpetersenii* serovar *Kenya*), *Canicola* (*L. interrogans* serovar *Canicola), Tarassovi (L. borgapetersenii* serovar *Tarassovi), Hebdomadis (L. borgapetersenii* serovar *Nona), Pomona (L. interrogans*serovar *Pomona), Shermani (L. santarosai* serovar *Shermani), Djasiman (L. interrogans* serovar *Djasiman), Pyrogenes (L. borgapetersenii serovar Nigeria), Sejroe (L. borgapetersenii* serovar *Sejroe), Icterohaemorrhagiae (L. kirschneri* serovar *Sokoline; L. interrogans* serovar *Icterohaemorrhagiae; L. interrogans* serovar *Copenhageni*), Grippotyphosa (*L. kirschneri* serovar Grippotyphosa).

Briefly, samples were retrieved from the refrigerator and they were run in two steps (screening and titration), first diluted in the ratio of 1:50 by adding 100 μl of test serum in 4.9 mls of s phosphate buffered saline (PBS) for screening. This preparation was screened in 96 well flat bottom microtitre plates with the panel of 14 live serovars. The first row of each plate was filled with controls serovars loaded in 50 μl of PBS and 50 μl of a live serovar making a final volume of 100 μl. The remaining rows were left for a single serum sample and each column for a single serovar. After loading of samples and test serovars, the plates were put into orbital plate shaker for 5 min for optimal mixing of sera and live serovars and then incubated for 2.5 h at 29 °C. The plates were examined by stereo microscope at high light intensity for agglutination^18^. Results were interpreted as positive and negative. Positive samples referred to those with a titre of ≥ 100.

### Data analysis

Data from the questionnaire was first entered in the research laboratory registers and later transferred into EPI DATA software package 2.1. Final analysis was done using R version 3.5.2 (2018-12-20). Prevalence was presented as percentages with respective confidence intervals. Prevalence was presented for the different patient characteristics (Table 1) including the species, serogroups and serovars (Table 2). A bivariable logistic regression was run where one independent variable was compared with the leptospirosis status (positive or negative) (Table 3). *p* values less than 0.05 were considered significant at 95% confidence interval. In addition, variables that had *p* value < 0.2 and those with biological and scientific significance were included in our multivariate logistic regression model. Adjusted odds ratio (AOR) at 95% Confidence Interval (CI) were incorporated in Table 4.

**Table 1:** Prevalence across patient characteristics table.

Variable	Level	Prevalence (%)	95% CI
Age	Below 10 years	9.10	[0.00–28.50]
	11–20 years	0.00	[0.00–10.60]
	21–30 years	7.30	[1.20–15.90]
	31–40 years	3.40	[0.40–12.30]
	Above 40 years	5.20	[3.10–15.30]
Sex	Male	5.60	[1.30–8.90]
	Female	3.90	[2.30–11.10]
Education level	Informal	5.60	[0.10–27.30]
	Primary	6.90	[2.60–14.40]
	Secondary	4.30	[1.20–11.10]
Occupation	Non-professional	4.30	[1.90–9.00]
	Professional	5.40	[2.00–12.80]
Contact with rodents	No	6.20	[3.10–10.80]
	Yes	1.30	[0.00–7.02]
Contact with animals	No	6.00	[2.60–11.50]
	Yes	3.30	[0.90–8.20]
Contact with wildlife	No	4.50	[2.30–7.80]
	Yes	14.30	[0.40–57.80]
Encountered floods	No	4.70	[2.20–8.70]
	Yes	4.90	[1.00–13.70]
Water source	Borehole	5.40	[0.70–18.30]
	Tap	5.30	[2.50–9.90]
	Well	2.10	[0.10–11.10]
	Tertiary	1.80	[0.00–9.60]
Fever	Yes	4.40	[1.20–9.10]
	No	3.90	[1.40–10.30]
Antibiotic use	Yes	0.00	[0.00–5.10]
	No	6.00	[2.70–9.20]

Where CI is confidence interval; professionals = all participants with a minimum of certificates in relation to their occupation; non-professionals = participants without a minimum of a certificate in relation to their occupation.

**Table 2:** Prevalence of leptospirosis by serovar table.

Species	Serogroup	Serovar	Percentage of seropositivity N = 14	Prevalence % (95% CI)
*L. interrogans*	Canicola	Canicola	14.30	0.70 (0.10–3.10)
*L. interrogans*	Icterohaemorrhagiae	Icterohaemorrhagia	7.10	0.40 (0.00–2.50)
*L. interrogans*	Pamona	Pamona	7.10	0.40 (0.00–2.50)
*L. interrogans*	Hebdomadis	Hebdomadis	14.30	0.70 (0.10–3.10)
*L. borgapetersenii*	Ballum	Kenya	14.30	0.70 (0.10–3.10)
*L. borgapetersenii*	Sejroe	Sejroe	14.30	0.70 (0.10–3.10)
*L. interrogans*	*Djasiman*	Djasiman	28.60	1.60 (0.50–4.20)

CI—stands for confidence interval.

**Table 3:** Bivariable analysis-table.

Variable	Level	UOR (95% CI)	*p* values
Age	Below 10	1	
	11–20	0.23 (0.01–0.51)	0.40
	21–30	0.31 (0.05–7.11)	0.65
	31–40	0.52 (0.06–6.31)	0.50
	41 and above	1.15 (0.18–5.10)	0.38
Sex	Female	1	
	Male	1.44 (0.38–5.94)	0.57
Education level	Primary and below	1	
	Secondary and above	0.49 (0.12–1.84)	0.24
Address	Central	1	
	Eastern	0.49 (0.12–1.84)	0.24
	Western	0.49 (0.12–1.84)	0.24
Occupation	Non-professional	1	
	Professional	1.27 (0.31–4.81)	0.76
Type of Domestic animals kept Cattle	No	1	
	Yes	0.26 (0.01–1.89)	0.30
Sheep	No	1	
	Yes	0 (0.00–5.64)	1
Goats	No	1	
	Yes	0.22 (0.005 1.56)	0.66
Fever	No	1	
	Yes	15.4 (1.17–150.57)	0.02
Dehydration	No	1	
	Yes	0.14 (1.00–1.0)	0.03
Antibiotic use	Yes	1	
	No	2.2 (0.37–9.7)	0.20
Abdominal pain	No	1	
	Yes	1.3 (0.33–5.5)	0.77

Where CI = confidence interval, UOR = unadjusted odds ratio, % = percentage, AOR = adjusted odds ratio.

**Table 4:** Multivariable logistic regression-table.

Variable	Level	Adjusted OR (95% CI)	*p* value
Age (years)	Below 10	1	–
	11–20	0.30 (0.01–8.71)	0.42
	21–30	0.23 (0.01 7.01)	0.34
	31–40	0.31 (0.02–8.23)	0.04*
	41 and above	0.42 (0.04–9.84)	0.50
Occupation	Non-professional	1	–
	Professional	1.95 (0.46–9.42)	0.22
Sex	Female	1	–
	Male	1.90 (0.54–7.45)	0.33
Dehydration	No	1	–
	Yes	0.10 (0.01–0.69)	0.05*
Abdominal pain	No	1	–
	Yes	24.40 (2.42–267.89)	0.02*

Where CI is confidence interval, OR is the odd ratio, *Significant at a *p*-value less or equal to 0.05.

### Ethics consideration

This study was approved by Mulago National Referral Hospital Research and Ethics Committee (approval number-MHREC 1347). In addition, written informed consent was sought from all the study participants before being recruited into the study. In circumstances were the participants involved minors (below 18 years old), assent was obtained from them while written informed consent was sought through the parent or guardian that escorted them to the health facility. We also followed the Helsinki declaration guidance on ethical principles of involving human subjects in this study^19^.

## Results

### Prevalence of leptospirosis

**Figure 1:**
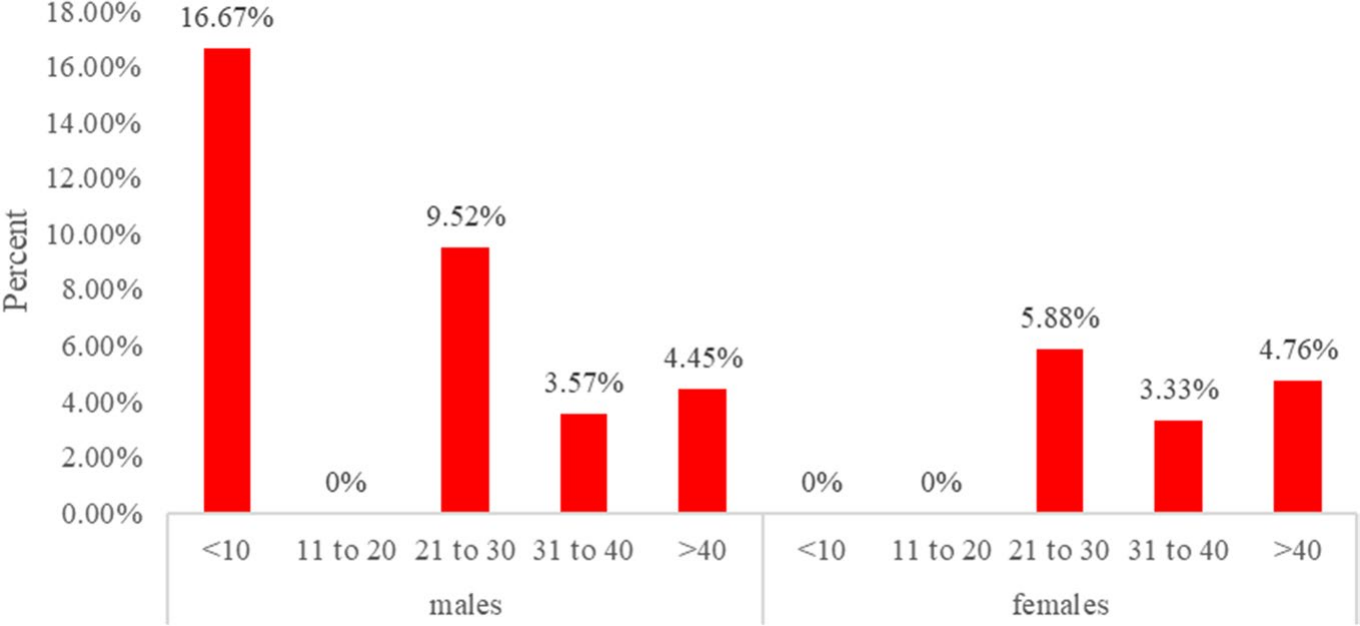
Age specific prevalence by gender of respondents.

The overall prevalence of leptospirosis was 4.70% (CI = 2.60–8.30) among all the patients recruited in this study. The renal and general outpatient presented a prevalence of 2.70% and 2.00% respectively. In terms of gender, leptospirosis in males was (5.60%) and (3.90%) females. Leptospirosis was highly prevalent among patients whose occupations were professional (5.40%) and non-professional (4.30%). Also, leptospirosis was more prevalent (9.10%) among children below 10 years (Fig. 1). There was no detectable leptospirosis among patients who had been previously on antibiotics treatment (Table 1).

**Figure 2:**
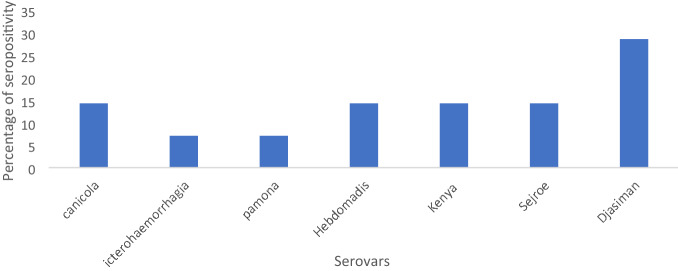
Prevalence of *leptospira* serovars.

In terms of seropositivity, a total of twelve (12) cases from both renal and general outpatient units were positive for *Leptospira* with titres ≥ 100. *Djasiman* and *Canicola* were more prevalent in both patient categories with titers of ≥ 200 and ≥ 400 respectively (Fig. 2). In the renal unit, one (1) individual was positive for both *Djasiman* and *Hebdomadis* serovars. Similarly, in the general outpatient unit, another individual was positive for *Icterhaemorhagic* strain RGA and *Canicola Leptospira* serovars (Table 2).

### Signs and symptoms associated with leptospirosis disease

Regarding signs and symptoms of patients, individuals who were dehydrated were less likely to be diagnosed with leptospirosis (AOR = 0.10, 95% CI (0.01–0.69), *p* = 0.05) (Table 4). Patients who reported with signs of abdominal pain were 24.4 times more likely to be diagnosed with leptospirosis (AOR = 24.40, 95% CI (2.42–267.89), *p* = 0.02) (Table 4).

## Discussion

This study investigated prevalence and associated signs and symptoms among patients who attended renal and general outpatient units of Mulago National Referral Hospital. Our study is the first to document leptospirosis status among the renal patients in Uganda. The findings from this study, revealed an overall prevalence of 4.7%. This prevalence was similar to that of 3.8% registered in northern Tanzania among hospitalized febrile patients^20^. A study conducted in south western Uganda among non-pregnant adult females attending lower health facilities however reported contrary results with leptospirosis prevalence at 35%^21^. Lower health facilities tend to offer antibiotics to non-malarial febrile patients and any other category that might be un-diagnosed. Since the *Leptospira* bacteria has been shown to be highly susceptible to prevailing antibiotics^22,23^, the prevalence goes low at each level of treatment even though the effects of the disease can be long term and often culminate into referrals. Therefore, previous exposure to antibiotics by patients attending referral hospitals such as MNRH could probably explain the observed low prevalence in this study. Lower health facilities should therefore enhance leptospirosis diagnosis in order to prevent progression of the disease to complicated phases of organ involvement such as kidney dysfunction. In addition, non-malarial febrile patients should be referred to health facilities with capacity to detect *Leptospira* in order to minimize symptomatic treatment which often leads to poor treatment outcomes.

*L. interrogans* serovar Djasiman was the main circulating pathogenic *Leptospira* serovar detected among seropositive individuals in this study. This serovar has not been reported before among humans in any healthcare facility-based study within Uganda. This Leptospira serovar despite having been reported elsewhere^24^, its high burden reported in this study could probably be due to the non-inclusion of the serovar in the testing panel in earlier studies^10,25^ or could be due to global travels since zero prevalence has overtime been reported among^26,27^. The high frequency of serovar Djasiman in this study suggested that a risk of developing severe leptospirosis exists in Uganda^24^. Other studies have implicated *L. interrogans* serovar Djasiman for causing massive alveolar hemorrhage^28^. The low prevalence of Icterohemorrhagiae in our study is in agreement with previous studies conducted on cattle^26,27^ and humans^10^ in Uganda. We report a moderate prevalence for *L. borgapetersenii* serova Sejroe. However, studies in Uganda and elsewhere have reported a high burden and severity of this serovar^12,29^. *L. borgapetersenii* serova Sejroe is *leptospira* associated bovine infection and its circulation in the human population indicates its zoonotic nature. Therefore, there is dire need to pay keen attention to the servars circulating within the country with particular emphasis on the ones with zoonotic potential.

Disease signs and symptoms such as fever, abdominal pain, dehydration, headache among others presented by patients at point of care are relevant in supporting clinical diagnosis of that particular health condition. Our study reveals that patients who reported with signs of abdominal pain were 24.4 times more likely to be diagnosed with leptospirosis. Abdomen pain among individuals diagnosed with leptospirosis was reported in a hospital based case study in Hawaii USA^30^. Patients attending the renal and general outpatient units with signs of dehydration were less likely to be diagnosed with leptospirosis. However other studies report contrary results due to the fact that leptospirosis infection is associated with fever and vomiting which are known to cause dehydration. Therefore, the observed dehydration among our study participants could probably be due to other disease conditions such as malaria, diarrhea among others.

## Conclusion

The prevalence of leptospirosis with previously unknown serovars among patients in Mulago National Referral Hospital of Uganda, indicates the burden and dynamics of the disease remains unknown in the country. This study suggests the need for a large-scale prospective cohort study in hospitals but more so the lower health facilities. The policy makers should advocate for inclusion of leptospirosis in the deferential diagnosis of febrile illnesses especially at lower level and private health facilities.

## Supplementary Information

Supplementary Information 1.

Supplementary Information 2.

Supplementary Information 3.
