# Supplementary material for: Prevalence of leptospirosis among patients attending renal and general outpatient clinics in Mulago Hospital, Kampala, Uganda
Source: Sci Rep. 2022 May 19;12:8391. doi: 10.1038/s41598-022-12544-3 (PMC9120167; doi:10.1038/s41598-022-12544-3)
Supplement: Supplementary file 1 — Supplementary Information 1. [file 41598_2022_12544_MOESM1_ESM.docx]

| **Introduction**  Good day,  My name is………………………………. I am conducting a study on the “**Prevalence of leptospirosis among patients attending renal and general outpatient clinics in Mulago hospital, Kampala, Uganda”.** The results of this study will be combined for all the respondents and individual details will not be revealed to anyone. This study will contribute to the understanding of the dynamics of infectious diseases like leptospirosis that are rarely targeted by health care workers. Your participation will be important to this study. In case you need more information, you can let me know and I can explain further. You are free to opt out in case you are not comfortable with anything. All answers will be correct.   1. Enroll me in the study (yes)………………(no)………………………….. 2. Enroll my child in the study (yes)………………(no)……………………. |
| --- |

| **PATIENT INFORMATION** | |
| --- | --- |
| **Age: Sex**: 􀁹Male 􀁹 Female **Occupation:** __________________  **Address**:  **Education level:** 􀁹Tertiary 􀁹 Secondary 􀁹 primary 􀁹 None  **Religion:**  **Contact #**:  **Hospital Hospital ID** # **Service** 􀁹Private􀁹charity􀁹government  **Case ID** # | |
| **CLINICAL INFORMATION** | |
| **Date of consult at hospital where questionnaire is being filled up** _ _/__/__ Day mo year  **Main complaint________ _ ______________Date of onset of symptoms**__/__/__Day mo year  **History of previous consult** 􀁹Yes 􀁹No  If YES Place________________ Diagnostics____________  **History of antibiotic treatment (within 4 weeks prior to consult)**  Have you been on treatment? 􀁹Yes 􀁹No  Could you be knowing the drugs you were using  Indication: 􀁹 treatment 􀁹 prophylaxis 􀁹 others_______________  If yes:  Medicines given (provide dose and duration)  􀁹 Penicillin G _________________ 􀁹 Ceftriaxone _______________  􀁹 Ampicillin__________________ 􀁹 Amoxicillin ______________  􀁹 Doxycycline________________ 􀁹 Azithromycin _____________ 􀁹 Hydrocortisone_____________ 􀁹 Methylprednisolone_____________ 􀁹 Prednisone _______________ 􀁹 Others______________  Dosage:___________ Duration________ | |
| **SYMPTOMS:** | |
| Fever 􀁹Yes 􀁹No  Malaise 􀁹Yes 􀁹No  Headech􀁹Yes 􀁹No  Chills 􀁹Yes 􀁹No  Muscle/joint pains 􀁹Yes 􀁹No  Diarrhea 􀁹Yes 􀁹No  Abdominal pain 􀁹Yes 􀁹No  Nausea/vomiting 􀁹Yes 􀁹No  Jaundice 􀁹Yes 􀁹No | Cough 􀁹Yes 􀁹No  Hemoptysis 􀁹Yes 􀁹No  Dyspnea 􀁹Yes 􀁹No  Decreased urine output 􀁹Yes 􀁹No  Hematuria/tea colored urine 􀁹Yes 􀁹No  Melena 􀁹Yes 􀁹No  􀁹Others______________________  ____________________________ |
| **PHYSICAL EXAMINATION:** | |
| Vital signs: Temp_______, BP______, HR_______, RR________ | |
| 1. Conjunctival suffusion 􀁹Yes 􀁹No 2. Jaundice 􀁹Yes 􀁹No 3. Signs of dehydration 􀁹Yes 􀁹No 4. Breath sounds 􀁹Yes 􀁹No | 1. Abdominal tenderness 􀁹Yes 􀁹No 2. Presence of wound/skin lesions􀁹Yes􀁹No 3. Edema 􀁹Yes 􀁹No 4. Others…….. |
| **CO MORBIDITIES** | |
| 1. Hypertension 􀁹Yes 􀁹No 2. Diabetes 􀁹Yes 􀁹No 3. Asthma 􀁹Yes 􀁹No 4. Liver disease 􀁹Yes 􀁹No | 1. Existing CKD 􀁹Yes 􀁹No 2. Malignancy 􀁹Yes 􀁹No 3. Others_______________________ |
| **EXPOSURE HISTORY** | |
| 1. History of exposure (working, swimming, submerged) in flood waters   􀁹None 􀁹Single 􀁹 Multiple   1. Date of first exposure__________________ 2. Place of exposure _____________________ 3. Contact with rodents, animal carcass, excreta, urine 􀂅 Yes 􀂅 No 􀂅 Don’t know 4. Do you own or in contact with any of these animals?   cattle 􀂅Yes 􀂅 No  Sheep 􀂅Yes 􀂅 No  Goats 􀂅 Yes 􀂅 No  Deer 􀂅 Yes 􀂅 No  Pigs 􀂅 Yes 􀂅 No  Dogs 􀂅Yes 􀂅 No  Cats 􀂅 Yes 􀂅 No  Other________________   1. Number of other members of household exposed 􀁹0 􀁹1 􀁹2 􀁹3 􀁹4 􀁹>4 2. Did you encounter flooding previously? 􀂅 Yes 􀂅 No 3. If yes when did it happen: ……………. 4. Where did you encounter flooding? 􀂅 Home 􀂅 on the way 􀂅 at work place 5. Did you have any footwear? 􀂅 Yes 􀂅 No 6. If yes which one of these? 􀂅 Gum boots 􀂅 sandals 􀂅 bare foot 􀂅 shoes 􀂅 others………… 7. Do you have any cut or abrasion on your body? 􀂅 Yes 􀂅 No 8. Which part of the body? 􀂅 Hand 􀂅 forearm 􀂅 leg 􀂅 foot 􀂅 others………. 9. Job description, please tick more than one if applicable:   􀂅 Veterinarian 􀂅 Medical worker􀂅 Driver 􀂅 Animal handler 􀂅 House helper 􀂅 Sewer worker 􀂅 waste manager􀂅 Teacher 􀂅others……….   1. Do you have any other work outside the occupation? 􀂅 Butcher 􀂅 Livestock farmer􀂅Non live stock 􀂅 None 􀂅others…… | |
| **PRACTICES AND BEHAVIORAL RISKS** | |
| 1. Do you wash your hands after? 􀂅 Yes 􀂅 No 2. When do you wash your hands? 􀂅 regularly 􀂅 whenever soiled 􀂅 rarely 3. What do you use in washing your hands? 􀂅 Water only 􀂅 water and soap 􀂅 sanitizer􀂅 others…. 4. Do you eat raw salads? Yes 􀂅 No 5. If yes when did you last eat salads: 􀂅 2 days ago 􀂅 1 week 􀂅 2 week 􀂅 1 month 􀂅 others…………. 6. Where do you get your water?   􀁹 Lake 􀁹 River 􀁹 Ponds 􀁹 Tap 􀁹 Well/spring􀁹 Bore hole 􀁹Others…….   1. How do you get rid of garbage at home? 􀂅 municipal waste managers 􀂅 household member 􀂅 myself 􀂅 others……… 2. Do you maintain your house? 􀂅 Yes 􀂅 No 3. If yes, what do you use for house maintenance: 􀂅 Water 􀂅cow dung 􀂅 cement and sand 􀂅 others…… 4. Do you have a latrine/toilet at home? 􀂅 Yes 􀂅 No | |
| **AWARENESS AND ATTITUDE TOWARDS ZOONOSES (LEPTOSPIROSIS)** | |
| 1. Are you aware of any disease that can be transmitted from animals to humans? 􀁹Yes 􀁹No 2. If yes, which of the following diseases are you aware of?  􀁹Leptospirosis brucellosis 􀁹 salmonellosis 􀁹tetanus 􀁹 rabies 􀁹 giardiasis 􀁹TB 􀁹others………. 3. Have you heard about leptospirosis? 􀁹Yes 􀁹 No 4. How is leptospirosis transmitted? 􀁹 by drinking un-boiled water 􀁹 by drinking raw milk 􀁹 by eating half cooked meat 􀁹 through a cut on the skin 􀁹through contact of contaminated urine of infected animal 􀁹 eating infected wild animals 􀁹others…… 5. What are the signs of leptospirosis in someone who is affected?  􀁹 Fever 􀁹headache 􀁹 backache 􀁹yellowing of the eyes 􀁹 joint pain 􀁹abdominal pain 􀁹diarrhea 􀁹vomiting 6. How can leptospirosis be prevented? 􀁹 through wearing of protective gears 􀁹 drinking boiled milk 􀁹through vaccination of animals 􀁹 drinking boiled water 􀁹 by washing hands 7. Does leptospirosis have a cure? 􀁹 Yes 􀁹No 􀁹 I don’t know 8. Can leptospirosis kill? 􀁹Yes 􀁹 No 􀁹 I don’t know | |
