# Supplementary figures and images for: Prevalence of leptospirosis among patients attending renal and general outpatient clinics in Mulago Hospital, Kampala, Uganda
Source: Sci Rep. 2022 May 19;12:8391. doi: 10.1038/s41598-022-12544-3 (PMC9120167; doi:10.1038/s41598-022-12544-3)

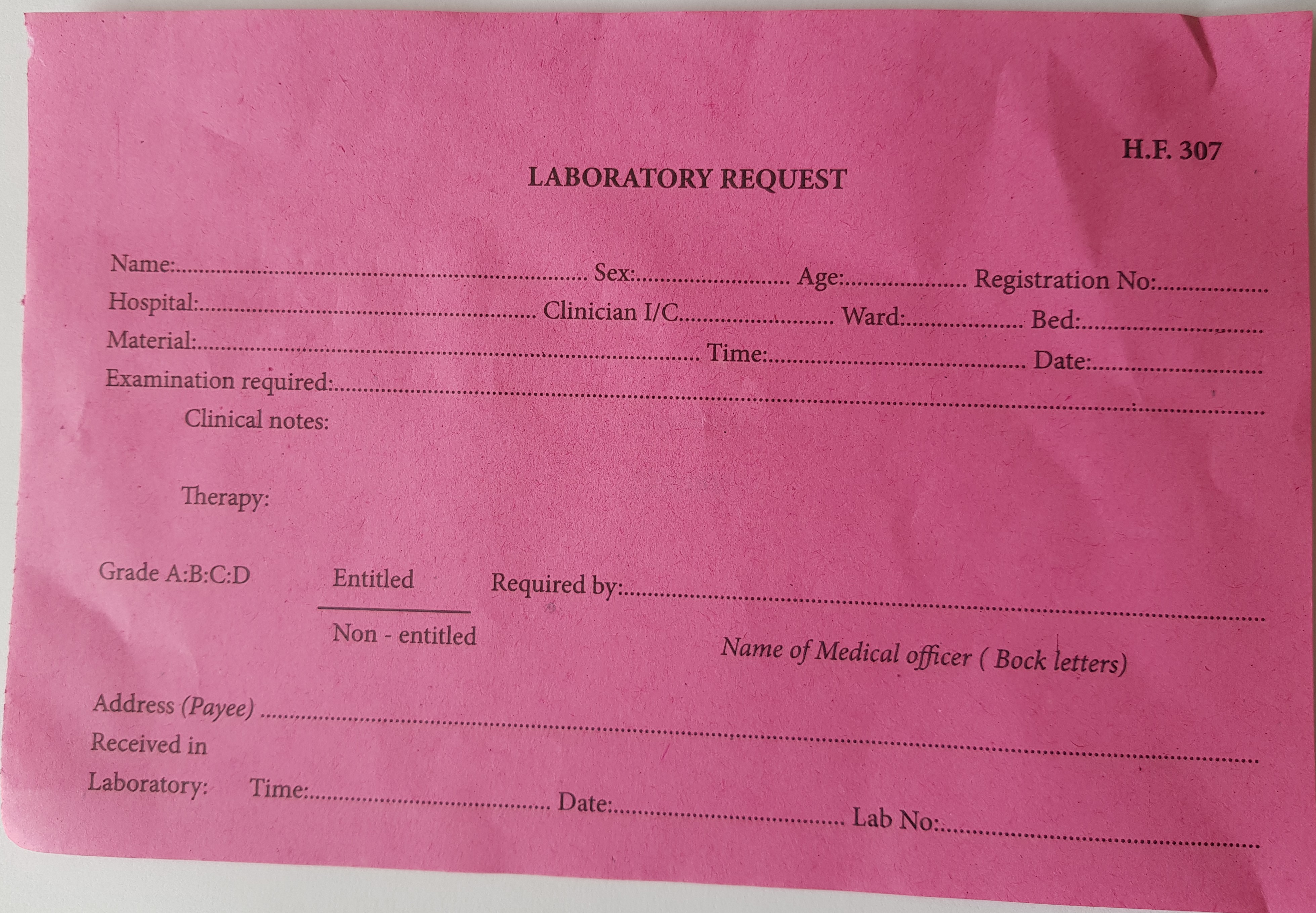

Supplement: Supplementary file 2 — Supplementary Information 2. [file 41598_2022_12544_MOESM2_ESM.jpg]
